# An analysis of pterosaurian biogeography: implications for the evolutionary history and fossil record quality of the first flying vertebrates

**DOI:** 10.1080/08912963.2014.939077

**Published:** 2015-07-28

**Authors:** Paul Upchurch, Brian Andres, Richard J. Butler, Paul M. Barrett

**Affiliations:** ^a^ Department of Earth Sciences, University College London, Gower Street, LondonWC1E 6BT, UK; ^b^ Department of Geology, University of South Florida, 4202 East Fowler Avenue, SCA528, Tampa, FL33630, USA; ^c^ School of Geography, Earth and Environmental Sciences, University of Birmingham, Edgbaston, BirminghamB15 2TT, UK; ^d^ Department of Earth Sciences, The Natural History Museum, Cromwell Road, LondonSW7 5BD, UK

**Keywords:** dispersal, diversity, pterosaur, sympatry, Treefitter, vicariance

## Abstract

The biogeographical history of pterosaurs has received very little treatment. Here, we present the first quantitative analysis of pterosaurian biogeography based on an event-based parsimony method (Treefitter). This approach was applied to a phylogenetic tree comprising the relationships of 108 in-group pterosaurian taxa, spanning the full range of this clade's stratigraphical and geographical extent. The results indicate that there is no support for the impact of vicariance or coherent dispersal on pterosaurian distributions. However, this group does display greatly elevated levels of sympatry. Although sampling biases and taxonomic problems might have artificially elevated the occurrence of sympatry, we argue that our results probably reflect a genuine biogeographical signal. We propose a novel model to explain pterosaurian distributions: pterosaurs underwent a series of ‘sweep-stakes’ dispersal events (across oceanic barriers in most cases), resulting in the founding of sympatric clusters of taxa. Examination of the spatiotemporal distributions of pterosaurian occurrences indicates that their fossil record is extremely patchy. Thus, while there is likely to be genuine information on pterosaurian diversity and biogeographical patterns in the current data-set, caution is required in its interpretation.

## 1. Introduction

**Figure 1:**
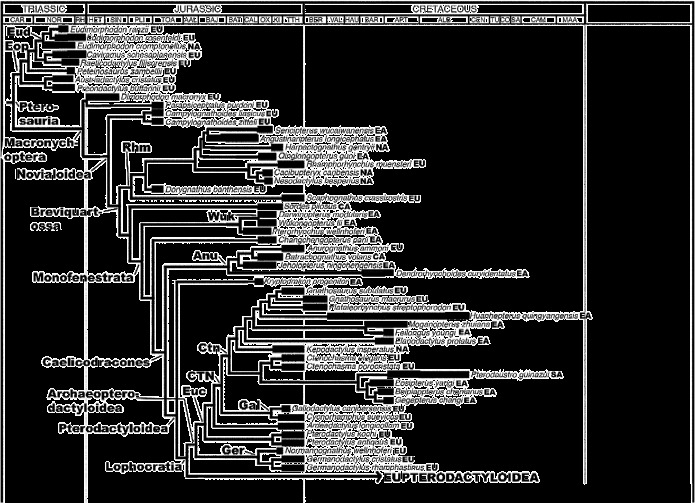
The pterosaur relationships and stratigraphical/geographical ranges used in the ‘all taxa’ Treefitter data-set. This tree is based on the cladogram presented by Andres et al. (2014) and shows the more basal portion in detail (Eupterodactyloidea has been condensed to a single branch – see Figure 2). The thick branches represent known stratigraphical ranges (based on data in *The Paleobiology Database*); thin branches represent estimated ghost ranges and connectors used to demarcate phylogenetic relationships. Time-sliced data-sets were derived from this tree by appropriate inclusion/exclusion of taxa. Most stratigraphical stage and taxon abbreviations are listed in the legend of Table 1. Additional abbreviations: CA, Central Asia; CO, Coniacian; EA, East Asia; Eop, Eopterosauria; EU, Europe; Euc, Euctenochasmatia; KI, Kimmeridgian; NA, North America; OX, Oxfordian; RH, Rhaetian; SA (after taxon name), South America; SA (time scale), Santonian; TU, Turonian.

**Figure 2:**
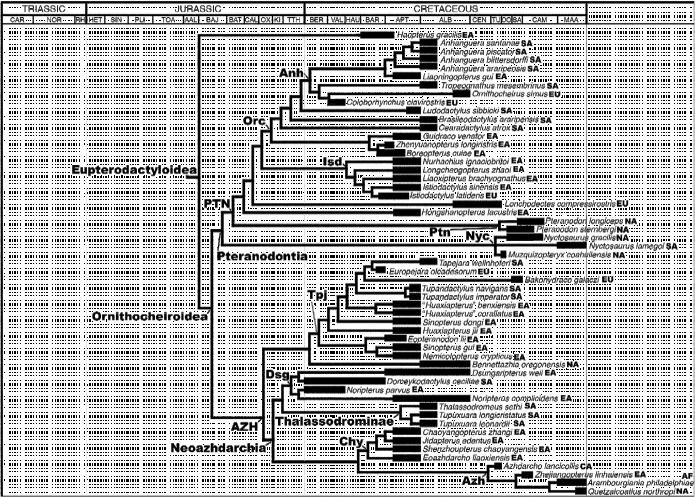
The pterosaurian relationships and stratigraphical/geographical ranges used in the ‘all taxa’ Treefitter data-set. This tree is based on the cladogram presented by Andres et al. (2014) and shows the relationships among Eupterodactyloidea (see Figure 1 for the more basal part of the cladogram). The thick branches represent known stratigraphical ranges (based on data in *The Paleobiology Database*); thin branches represent estimated ghost ranges and connectors used to demarcate phylogenetic relationships. Time-sliced data-sets were derived from this tree by appropriate inclusion/exclusion of taxa. All abbreviations are listed in the legend of Table 1 and/or Figure 1.

After their origin in the Middle or Late Triassic, pterosaurs acquired a virtually global distribution and their remains are now known from every continent, including Antarctica (Barrett et al. 2008; see *Fossilworks* and *The* *Paleobiology Database*). As with dinosaurs and many other clades, pterosaurian evolution took place against a backdrop of profound changes in palaeogeography driven by the fragmentation of Pangaea, major fluctuations in sea level and shifts in climatic zones. It is therefore surprising that there has been very little detailed study of pterosaurian biogeographical history (though see Unwin 1996; Wang et al. 2005, 2007, 2012). This neglect may reflect the intense focus on the flight mechanics of these organisms, and/or the implicit assumption that the geographical distributions of flying organisms are affected more by specific ecological requirements rather than large-scale vicariance and coherent dispersal patterns. In this paper, we present the first detailed analytical study of pterosaurian biogeographical history. First, we provide an overview of the pterosaurian fossil record, summarising where and when particular clades are represented and adding further information based on ghost ranges. Second, we briefly review the small number of previous studies that have proposed hypotheses to account for aspects of the spatiotemporal distributions of pterosaurs. Third, we test these and other hypotheses by applying a cladistic biogeographical analysis using Treefitter 1.2b (Ronquist 1998; Sanmartin and Ronquist 2004), to a recent phylogeny for pterosaurs (Andres et al. 2014) termed here the ‘reference phylogeny’ (Figures 1 and 2), in order to determine whether there is any statistical support for particular distribution patterns. Such analyses also enable an assessment of the relative importance of processes such as vicariance, dispersal, extinction and sympatric speciation in pterosaurian evolution. Finally, we end with a brief discussion of the quality of the pterosaurian fossil record and future requirements and prospects for further work on the biogeographical history of this clade.

## 2. Pterosaurian distributions through space and time

**Figure 3:**
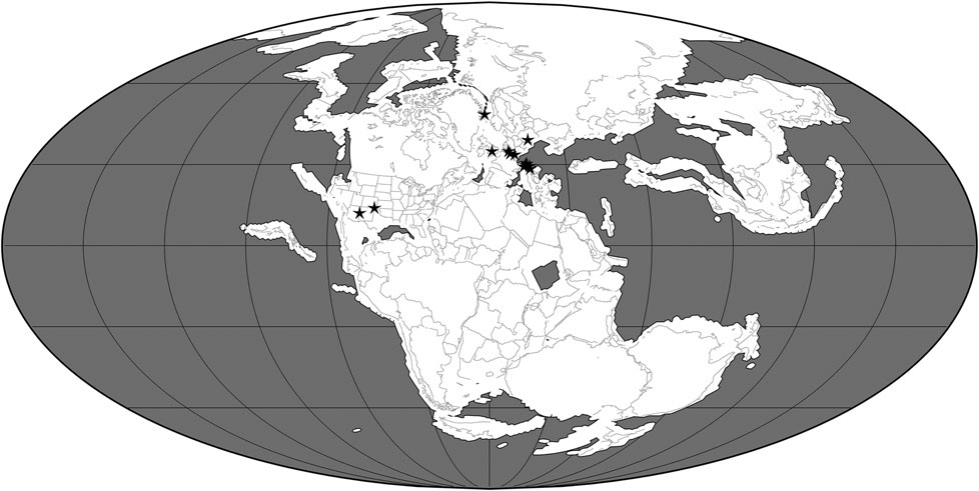
Palaeogeographical map for the Late Triassic (210 Ma) showing the locations of 29 collections of pterosaurian specimens. The map was generated using software available at Fossilworks (Alroy 2013), with collections data downloaded from The Paleobiology Database.

Below, we use the atlas of pterosaurian distributions by Barrett et al. (2008) (with revisions based on *The Paleobiology Database* (http://paleobiodb.org/#/), Fossilworks (http://fossilworks.org/) and Brian Andres, pers. obs.) to generate an overview of this group's spatiotemporal distribution (Figures 3 4 5 6 7, Table 1). This review provides a framework for the analyses that follow and also raises several issues that we believe should be addressed by future studies. The reader should note that there are some inconsistencies between the various classifications of pterosaurs applied by Barrett et al. (2008) and in *Fossilworks* and *The Paleobiology Database* and the reference phylogeny (Figures 1 and 2) employed here in the Treefitter analyses. Here, we have employed pterosaurian group names and taxonomic contents that are consistent with the phylogeny presented by Andres et al. (2014).

**Figure 4:**
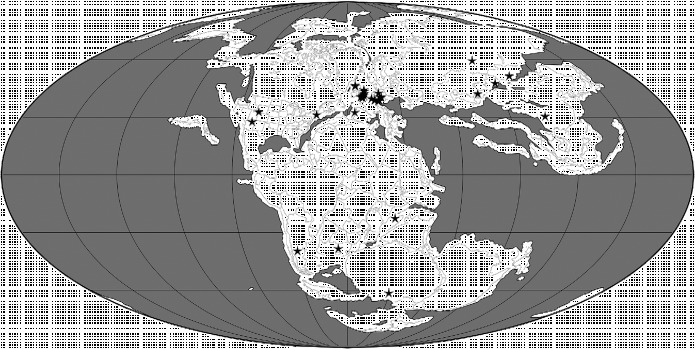
Palaeogeographical map for the Early and Middle Jurassic (170 Ma) showing the locations of 88 collections of pterosaurian specimens. The map was generated using software available at Fossilworks (Alroy 2013), with collections data downloaded from The Paleobiology Database.

**Figure 5:**
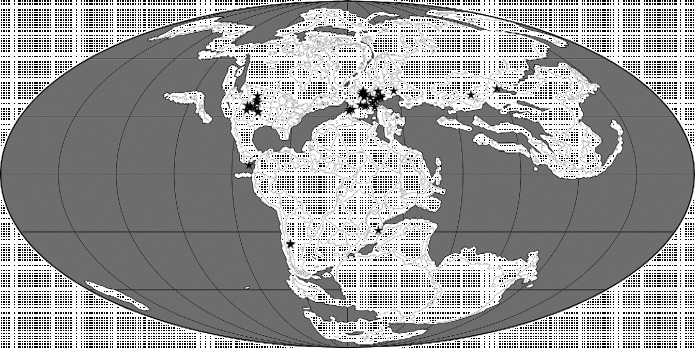
Palaeogeographical map for the Late Jurassic (150 Ma) showing the locations of 77 collections of pterosaurian specimens. The map was generated using software available at Fossilworks (Alroy 2013), with collections data downloaded from The Paleobiology Database.

**Figure 6:**
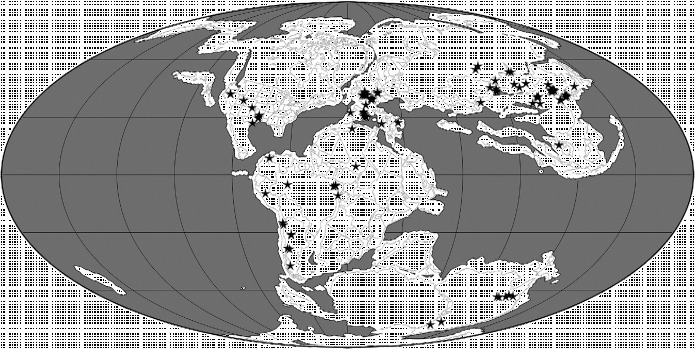
Palaeogeographical map for the Early Cretaceous (130 Ma) showing the locations of 176 collections of pterosaurian specimens. The map was generated using software available at Fossilworks (Alroy 2013), with collections data downloaded from The Paleobiology Database.

**Figure 7:**
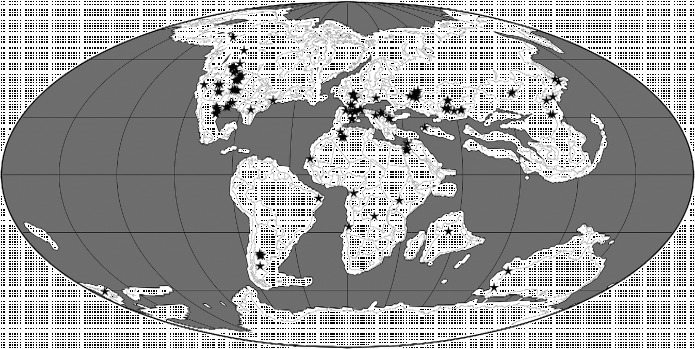
Palaeogeographical map for the Late Cretaceous (80 Ma) showing the locations of 182 collections of pterosaurian specimens. The map was generated using software available at Fossilworks (Alroy 2013), with collections data downloaded from The Paleobiology Database.

**Table 1:** A summary of the geographical and stratigraphic distributions of pterosaur families and other major clades (based on Barrett et al. 2008 modified by Andres, personal data).

Stage	Africa	Antarctica	Australia	Central Asia	East Asia	Europe	India	North America	South America
Car								Eud	
Nor						Aus, Eud, Pre, Pte, Pti			
Rha						Pti			
Het						Dmp, Pti			
Sin			Dmp			Dmp		Pti	
Pli	Pti						Pti	Pti	
Toa						Cmp, Dmp, Rhm,			
Aal					Pti			Pti	
Baj				Rhm	Rhm				
Bat	Pti			Pti	Anu, Pto, Rhm	Pti, Rhm	Pti	Pti	
Cal				Rhm	Anu	Pti, Rhm		Pti	Rhm
Oxf				Anu, Pdi, Rhm, Sor	Rhm, Wuk	Pti		Rhm	
Kim	Ctn, Pdi, Pti, Ten					Ard, Gal, Ger, Pti, Ptl, Rhm		Ctn, Pdi, Pti, Rhm	
Tth					Pti	Anu, Ctn, Gal, Ger, Pdi, Pti, Ptr, Rhm			Pdi
Ber	Ctn, Pti				Ctn, Dsg, Pti	AZH?, Ctn, Lnc, Orc, Pdi, Pti			Ctn, Dsg
Val					Ctn, Orc, Pdi, Pti	Orc			
Hau				Orc	Pti	Orc, Pti, PTN			Pdi
Bar				Orc	Anu, Ctn, Hao Orc, Tpj	Isd, Orc, Pdi, Pti, Tpj			Anh
Apt	AZH, PTN			Orc	Anh, Chy, Ctn, Dsg, Isd, Orc, PTN, Tpj,	Pti, PTN		Azh, PTN, Pdi, Pti	Anh, AZH, Ctn, Orc, Pti, Tpj
Alb	Orc		Orc, Pti,	Pdi	PTN	Anh, Azh, Lnc, Orc, Pti		AZH, Azh	Anh, Ctn, Orc, PTN, Pti, Tpj
Cen	Anh, Azh, Orc, Pdi, Tpj			Azh, Orc, Pdi, PTN	Azh, Pdi	Anh, Lnc, Orc, Pti		Pti, Ptn	AZH
Tur				Azh		Pdi		PTN	Pdi
Con						AZH,		Nyc, Ptn	
San				Azh, Pti	Azh, Pti	Azh, Tpj		Nyc, Pti, Ptn	
Cam	Azh, Pti		Pdi	Azh, PTN	Azh, Pdi	Azh, Pdi, Pti		Azh, Nyc, Pdi, Pti, Ptn	Azh
Maa	Azh	Pdi		Azh		Azh, Pti	Pti	Azh, Pti	Nyc

Notes: Clade names used here are based on those in the reference cladogram in Figures 1 and 2, and therefore often differ in precise definition or content from those used by Barrett et al. (2008). When there is uncertainty in the age of the earliest known member of a taxon, it is shown in the oldest possible stage**.** Stratigraphic stage abbreviations: Aal, Aalenian; Alb, Albian; Apt, Aptian; Baj, Bajocian; Bar, Barremian; Bat, Bathonian; Ber, Berriasian; Cam, Campanian; Car, Carnian; Can, Cenomanian; Con, Coniacian; Hau, Hauterivian; Het, Hettangian; Kim, Kimmeridgian; Maa, Maastrichtian; Nor, Norian; Ox, Oxfordian; Pli, Pliensbachian; Rha, Rhaetian; San, Santonian; Sin, Sinemurian; Toa, Toarcian; Tth, Tithonian; Tur, Turonian; Val, Valanginian. Taxonomic abbreviations: Anh, Anhangueridae; Anu, Anurognathidae; Ard, *Ardeadactylus* *longicollum*; Aus, *Austriadactylus*; Azh, Azhdarchidae; AZH, Azhdarchoidea; Chy, Chaoyangopteridae; Cmp, *Campylognathoides;* Ctn, Ctenochasmatidae; Dmp, *Dimorphodon* and potentially closely related material; Dsg, Dsungaripteridae; Eud, *Eudimorphodon*; Gal, Gallodactylidae; Ger, Germanodactylidae; Hao, *Haopterus*; Isd, Istiodactylidae; Lnc, Lonchodectidae; Nyc, Nyctosauridae; Orc, ‘Ornithocheiridae’ *sensu* *lato*; Par, *Parapsicephalus*; Pdi, indeterminate pterodactyloid remains; Pre, *Preondactylus*; Pte, *Peteinosaurus*; Pti, indeterminate pterosaur remains (includes trackways); Ptn, Pteranodontidae; PTN, Pteranodontoidea; Pto, *Pterorhynchus*; Ptr, Pterodactylidae; Rhm, Rhamphorhynchidae; Sor, *Sordes*; Ten; Tendaguripteridae; Tpj, Tapejaridae *sensu lato*. Wuk, Wukongopteridae.

### 2.1 Middle and Late Triassic

The sister taxon to Pterosauria within Ornithodira, the Dinosauromorpha, has its earliest known body fossils in deposits of Anisian age (Nesbitt et al. 2010, 2013), and trackways suggest that this clade dates back to the early Olenekian (Brusatte et al. 2011) (Figure 3). This implies that the pterosaurian lineage was also present in the Middle Triassic, although the oldest body fossils of this clade are Carnian in age (see below). Previous phylogenetic analyses of basal pterosaurs imply the existence of at least three lineages during the Late Triassic (Andres et al. 2010) and as many as seven (Kellner 2003; Wang et al. 2008); however, the reference phylogeny used here (Figure 1) supports the existence of only one major ghost range during this interval. Minimally, body fossils and ghost ranges indicate that members of both the Macronychoptera and Eopterosauria were present as early as the Carnian (Figure 1), although no Triassic fossils belonging to the former clade have been found to date. Thus, pterosaurs almost certainly had a pre-Carnian origin. The first pterosaurian remains are known from strata of probable late Carnian–early Norian age in North America and include material assigned to *Eudimorphodon* (Murry 1986; Lucas and Luo 1993; Andres 2006; Figure 3, Table 1). Other pterosaurian remains have been reported from Carnian and Norian sediments in this region, but these specimens are indeterminate (e.g. Hunt and Lucas 1993) and cannot be confirmed as pterosaurs (Andres 2006). Similarly, Bonaparte et al. (2006) reported pterosaurian remains from the Carnian of Brazil, but the affinities of this material remain poorly understood and it is not certain that it represents a true pterosaur (Dalla Vecchia 2013) and it has recently been reinterpreted as a basal ornithodiran (Soares et al. 2013). The only other occurrences of pterosaurs in the Late Triassic are records from the Norian and Rhaetian of Greenland and Europe. The latter include *Preondactylus*, *Austriadactylus*, *Caviramus*, *Raeticodactylus*, *Peteinosaurus* and species of *Eudimorphodon* (Wild 1978, 1994; Dalla Vecchia 2003a, 2003b, 2003c; Fröbisch and Fröbisch 2006; Stecher 2008). It could be argued that this early fossil record suggests that pterosaurs originated in northwestern Pangaea (Euramerica) during the late Middle Triassic, and this is supported by the observation that the three-most basal lineages in Figure 1 (i.e. Eopterosauria, *Dimorphodon*+ *Parapsicephalus* and *Campylognathoides*) comprise almost exclusively European taxa and a few remains from Greenland and North America (Figure 3). However, the absence of pterosaurs from the rest of the world during the Late Triassic might merely reflect sampling biases (Butler et al. 2009). Moreover, given that the earliest dinosauromorphs and basal members of major dinosaurian clades are typically Gondwanan (e.g. Langer 2004; Nesbitt et al. 2010, 2013), there must be considerable uncertainty about the true geographical range of the pre-Carnian pterosaurian lineage and the extent to which the ornithodiran clade as a whole had achieved a global distribution prior to the dinosauromorph–pterosaur split.

### 2.2 Early and Middle Jurassic

Early Jurassic pterosaurian specimens have been reported from Africa (but not described; Blackbeard and Yates 2007), Antarctica (Hammer and Hickerson 1994), India (Rao and Shah 1963), Europe (Buckland 1829) and North America (Padian 1984), although they are currently unknown from Central and East Asia (Table 1; Figure 4). It therefore seems probable that pterosaurs had achieved, or were on their way to achieving, a virtually global distribution by the Early Jurassic (Figure 4). During this epoch, pterosaurs were best represented in Europe where *Campylognathoides* and Rhamphorhynchidae appear in the Toarcian fossil record (Newton 1888; Padian 2008). Members of the more basal ‘Dimorphodontidae’ (i.e. *Dimorphodon* itself and material said to be very similar to it, although this identification has yet to be confirmed by a detailed study) appear even earlier (Hettangian possibly, but more probably Sinemurian) and are known from the palaeogeographically widely separated regions of Europe (Padian 1983) and Antarctica (Hammer and Hickerson 1994) during this epoch (Table 1). Thus, the Macronychoptera were present by the Hettangian at the latest, and had diversified into ‘Dimorphodontidae’, *Campylognathoides* and Rhamphorhynchidae by the Toarcian (Figure 1). The latter two clades might have originated in Europe, but their absence from the rest of the world during the Early Jurassic might reflect a sampling bias given the general scarcity of pterosaurian remains during this time interval.

In the Middle Jurassic, pterosaurs are known from all regions except Antarctica and Australia (Table 1). Rhamphorhynchids were still present in Europe, and also appear for the first time in the fossil records of Central Asia (Nessov 1990; Averianov et al. 2005; Martin et al. 2006), East Asia (He et al. 1983) and South America (Rauhut et al. 2001; Codorniú and Gasparini 2007) (Table 1). The stratigraphically earliest pterodactyloid had been reported to be of Callovian age and was collected from deposits in Central Asia (Andres and Clark 2005; Clark et al. 2006), but recent recalibrations and the time scale of Gradstein et al. (2012) have altered the dating of this specimen to the earliest Late Jurassic within the error margin of the Middle–Late Jurassic boundary. A pterodactyloid has been reported from the Daohugou locality, Inner Mongolia (Ji and Yuan 2002), along with the earliest anurognathids (Wang et al. 2002) and a rhamphorhynchid (Czerkas and Ji 2002) now considered a basal monofenestratan. However, Ji and Yuan (2002) report a short-tailed pterodactyloid and a long-tailed rhamphorhynchoid, but only figure a short-tailed anurognathid pterosaur later referred to *Jeholopterus ningchengensis* (Lü 2009). It is most likely that this undescribed pterodactyloid is the figured anurognathid. The dates of these East Asian deposits are very controversial and have been identified as Middle Jurassic, Late Jurassic and/or Early Cretaceous in age (Swisher et al. 1999; Gao and Ren 2006; Xu and Hu 2009). Here, we provisionally accept an Oxfordian age suggested by U–Pb SHRIMP dates reported by Liu et al. (2012) (see also Sullivan et al. 2014). A Middle Jurassic specimen from Central Asia has been said to be similar to the anurognathids (Bakhurina and Unwin 1995; Unwin and Bakhurina 2000), but it does not preserve apomorphies of this group (Brian Andres, pers. obs.). Thus, the fossil record and ghost range reconstructions (Figure 1) suggest that the Monofenestrata appeared during the Middle Jurassic (probably prior to the Bathonian) and had diversified into the Wukongopteridae, Anurognathidae and Pterodactyloidea by the end of this epoch. Pterodactyloids and anurognathids might have originated in East and/or Central Asia, but their absence from the rest of the world during the Middle Jurassic could also be the result of poor sampling.

### 2.3 Late Jurassic

The European anurognathid *Anurognathus ammoni* is currently known from the Late Jurassic (Figure 5). This clade persisted beyond the Jurassic–Cretaceous boundary and is found in the Early Cretaceous of East Asia (e.g. Gao et al. 2009; Chang et al. 2014), but at present is not known from any Gondwanan continent or North America (Table 1) (note that the holotype of *Mesadactylus ornithosphyos* from the Late Jurassic of North America has been suggested to be an anurognathid [Bennett 2007], but it is not referred to this group here because the sole character used to ally it with this clade [relatively thinner first sacral ribs] is present in many other pterosaurs [Brian Andres, pers. obs]). The pterodactyloid clades Ctenochasmatidae, Gallodactylidae, Germanodactylidae, Pterodactylidae and Tendaguripteridae appear for the first time in the Kimmeridgian or Tithonian (Table 1), suggesting that the divergence of the archaeopterodactyloid and eupterodactyloid radiations occurred during or before the Oxfordian (Figure 1). In the Late Jurassic, the pterodactylids, gallodactylids and germanodactylids are only known from Europe and are considered to be basal archaeopterodactyloid clades in the current phylogenetic analysis. The Tendaguripteridae is known from a single specimen in Africa and has not been included in any phylogenetic analysis. The earliest ctenochasmatid pterosaurs have been found in North America, Europe and Africa in Kimmeridgian and Tithonian deposits (Wellnhofer 1978; Bennett 1995, 1996a, 1996b; Figure 1). A reported Late Jurassic ctenochasmatid from East Asia, *Huanhepterus quingyangensis* (Dong 1982), has since been re-dated as Early Cretaceous (Wang and Lü 2001). By the Early Cretaceous, this clade was also present in South America (Martill et al. 2006; see below). Dsungaripterid and azhdarchid specimens have been reported from the Late Jurassic of Europe. However, these referrals are based on character states that are widespread in pterosaurs and there is more character data that support placement in other clades (Andres and Ji 2008; Brian Andres, pers. obs.). For example, the putative Late Jurassic European azhdarchid material has been shown to belong to the contemporaneous Ctenochasmatidae by the phylogenetic analysis of Andres and Ji (2008), and material from Solnhofen recently identified as azhdarchid by Frey et al. (2011) has since been referred to the Ctenochasmatidae (Bennett, 2013). Isolated cervical vertebrae from the Tendaguru Beds of Tanzania in Africa have also been referred to the Azhdarchidae (Kellner et al. 2007; Costa et al. 2014). However, when subjected to phylogenetic analysis, these vertebrae were placed in the contemporaneous Ctenochasmatidae, which have similar cervical vertebrae (Andres and Ji 2008). Fragments from the Upper Jurassic of Shandong, China, referred to the Dsungaripteridae by Young (1964), also cannot be confirmed as belonging to this group (Brian Andres, pers. obs.).

### 2.4 Early Cretaceous

Isolated teeth from the Berriasian of Morocco have been proposed as putative early ‘ornithocheirids’, but these teeth are more similar to those found in the rhamphorhynchids *Sericipterus* and *Angustinaripterus* (Andres et al. 2010) (Figure 6). Thus, the earliest confirmed ornithocheiroids (derived pterodactyloids including members of the Pteranodontoidea and Azhdarchoidea [*sensu* Kellner 2003]) are instead the ‘ornithocheirid’ *Coloborhynchus clavirostris* and the dsungaripterid *Noripterus* *parvus* dated to the Berriasian–Valanginian (Figure 2) [note that Martill et al. (2013) reported a ‘possible azhdarchid’ metacarpal from the Berriasian of the UK, but this specimen is provisionally regarded as Azhdarchoidea? indet. here – see Table 1]. These records indicate that the ornithocheiroids had originated by the earliest Cretaceous (but probably diverged somewhat earlier in the Late Jurassic) and that this group had already diversified into several lineages (e.g. Anhangueridae, Istiodactylidae, Tapejaridae, Dsungaripteridae and Neoazhdarchia) by the Valanginian at the latest. Given that body fossils pertaining to these various ornithocheiroid clades do not appear until the Barremian (e.g. istiodactylids) or even later, it seems that the early Early Cretaceous fossil record of pterosaurs is particularly poor, in contrast to the rich Lagerstätten from China and Brazil of late Barremian–Aptian and Albian age, respectively (see below). The ornithocheiroid clade seems to have achieved a virtually global distribution (Africa, Europe, South America, East Asia and Central Asia) by the Barremian or Aptian, and it even provides a rare pterosaurian record from the Albian of Australia (Molnar and Thulborn 1980; Table 1). If this widespread distribution of ornithocheiroid clades evolved during the Early Cretaceous, this would have required dispersal across several marine barriers [e.g. the proto-North Atlantic, Gulf of Mexico/Caribbean corridor, Pacific Ocean and/or Turgai sea; see Smith et al. (1994) and Scotese (2004) for palaeogeographical reconstructions].

Lonchodectid pterosaurs represent a minor radiation that was apparently restricted to the Early Cretaceous (Berriasian–Cenomanian) of Europe. This clade has also been tentatively identified in the Albian of Australia (Molnar and Thulborn 2007), but more recently this material has been provisionally regarded as closely related to, but not a member of, the Anhangueridae (Kellner et al. 2011) (Table 1).

### 2.5 Late Cretaceous

From the Cenomanian to the Maastrichtian, pterosaurs maintained a global distribution, but their diversity was apparently somewhat lower (Butler et al. 2009, 2012, 2013) (Figure 7). This reduced Late Cretaceous diversity partially reflects the putative extinction of several clades (e.g. anhanguerids, ctenochasmatids, istiodactylids and lonchodectids) by the end of the Cenomanian (Table 1). Thus, pterosaurs might have been affected by the spatially and temporally staggered series of turnover events in the mid-Cretaceous noted by Benson et al. (2013) that transformed Late Cretaceous dinosaurian, crocodyliform, mammalian and lepidosaurian faunas. As a result, Late Cretaceous pterosaurian faunas are almost exclusively composed of members of the Pteranodontidae, Nyctosauridae and Azhdarchidae. This apparent pattern of mid-Cretaceous faunal turnover among pterosaurian groups might reflect clade–clade interactions between pterosaurs and birds, especially as members of the latter clade diversified to occupy many small and medium body-size niches (Benson et al. 2014). However, the comparative scarcity of Late Cretaceous Lagerstätten for these groups (Butler et al. 2009; Brocklehurst et al. 2012) means that apparent decreases in pterosaurian diversity, occurring just after the rich Aptian and Albian faunas of China and Brazil, should be treated with caution. While it seems highly probable that a decrease in pterosaurian diversity during the Late Cretaceous is a real phenomenon (Butler et al. 2009, 2012, 2013), the precise timing and rate of extinction events have probably been distorted by uneven sampling.

At present, confirmed pteranodontids are only known from the Late Cretaceous of North America. Nyctosaurids are similarly restricted, although one species also occurs in South America (Price 1953; Table 1). In contrast, although their diversity is low, azhdarchids were globally distributed in the latest Cretaceous, occurring in Africa, Central and East Asia, Europe and North America, and have just been reported for the first time from South America (Novas et al. 2012).

## 3. Previous studies of pterosaurian biogeography

Very little attention has been paid to the biogeographical history of pterosaurs, and consequently, there are few explanatory hypotheses in the literature pertaining to their observed distribution in the fossil record. Below, we briefly summarise the biogeographical hypotheses proposed by such studies of pterosaurs, and some relevant ideas derived from work on other Mesozoic groups such as dinosaurs. These hypotheses are examined in the light of our current knowledge of pterosaurian phylogenetic relationships and the quality of this group's fossil record.

Unwin (1996, p. 300) stated that ‘…it is not until the Middle Jurassic…that pterosaurs are known from virtually all major land masses’. Discoveries during the past 15 years have substantially broadened the Early Jurassic distribution of pterosaurs (see above, Table 1). It seems probable that pterosaurs were more widespread in the Early Jurassic than previously realised, but they might not have been truly globally distributed and abundant until the Middle Jurassic (Barrett et al. 2008).

Several authors have suggested that Central and East Asia were geographically isolated from the rest of Pangaea by the epicontinental Turgai sea during the Middle and Late Jurassic (Russell 1993; Upchurch et al. 2002; Wilson and Upchurch 2009), and it is also possible that the Mongol–Okhotsk Sea between Siberia–Kazakhstan and Mongolia–China produced an additional barrier between Central and East Asia (Upchurch 1995). The first pterodactyloids were present in Central Asia but were apparently absent from Europe – a pattern that is consistent with the East Asian isolation model. However, if the Turgai and/or Mongol–Okhotsk seas prevented the dispersal of early pterodactyloids to regions outside of Central Asia, then we would have to explain why these barriers did not prevent the apparent dispersal of rhamphorhynchids from Europe to Central Asia at this time. One possibility is that the Pterodactyloidea originated as an exclusively terrestrial group, unlike rhamphorhynchids that are found in both marine and terrestrial settings (Lü et al. 2010). To date, only one quantitative analysis of environmental preferences among pterosaurian clades has been attempted (Andres et al. 2014): this found evidence for a preference for terrestrial environments among pterodactyloids. As with many other palaeobiogeographical scenarios, it is difficult to determine to what extent the absence of European Middle Jurassic pterodactyloids reflects poor sampling versus genuine absence. European Middle Jurassic sediments have produced other pterosaurs such as rhamphorhynchids, but these specimens are very fragmentary: it is therefore possible that pterodactyloid material has either not been recovered or has not been recognised because of its highly incomplete preservation. Thus, although it is conceivable that geographical isolation of parts of Asia played a role in the origin of an initially endemic pterodactyloid clade, it would be premature to rule out the possibility that pterodactyloids were actually widespread at this time but have their true distribution obscured by very poor sampling.

Confirmed istiodactylid ornithocheiroids are only known from Europe (Barremian) and eastern Asia (Aptian). Such a distribution is consistent with the Aptian geodispersal event proposed by Russell (1993), Norman (1998) and Upchurch et al. (2002) in which several dinosaur lineages and other terrestrial taxa apparently dispersed from Europe to Asia (or vice versa) as a result of a land bridge across the Turgai sea produced by marine regression (see also Barrett et al. 2002; Wilson and Upchurch 2009). It should be noted that *Istiodactylus latidens* from Europe is not only the earliest of the known istiodactylids but also one of the most derived (Figure 2). Thus, while the phylogenetic relationships are consistent with dispersal from East Asia to Europe, this is not well supported stratigraphically. *Istiodactylus* is not the only pterosaur to have been implicated in a possible geodispersal event between Europe and East Asia during the Early Cretaceous. Wang et al. (2005, p. 877) noted that *Feilongus* and *Nurhachius*, from the Jehol Group, were most closely related to European taxa, and suggested that this supported hypotheses of faunal exchange between Asia and Europe during the Early Cretaceous. Although the relationships of these taxa are different in Figure 1, and their closest relatives are also from East Asia, *Feilongus* and *Nurhachius* still cluster with European taxa. This biogeographical scenario was reinforced by Wang and Zhou (2006) and Wang et al. (2007) who argued that representatives of the clades Anurognathidae, Rhamphorhynchidae, Gallodactylidae, Ornithocheiridae, Pterodactylidae and Ctenochasmatidae were present in the Late Jurassic of Europe but absent from Asia until the Early Cretaceous. These authors inferred one or more dispersal events from Europe to East Asia, during the Early Cretaceous, as a result of the disappearance of geographical barriers. However, Wang et al. (2005, p. 877), citing Zhou et al. (2003), also cautioned that other Jehol pterosaurs appear to be most closely related to Brazilian taxa, and that the ‘…palaeobiogeographic history of the Jehol biota is very complex’.

Wang and Zhou (2006) proposed that western Liaoning was the centre of origin for the clades Anhangueridae and Tapejaridae, based on the observation that the oldest known members of these clades are from this area. Subsequently, Wang et al. (2008) suggested that Asia might represent a centre of origin for derived ornithocheiroid pterosaurs, based on the description of the Jehol taxon *Nemicolopterus* (considered by them to be the sister taxon to Ornithocheiroidea, but here recovered as a tapejarid; Figure 2). Wang et al. (2012) proposed that *Guidraco* from China and *Ludodactylus* from Brazil are sister taxa and therefore argued for close biogeographical links between these two areas in the Aptian–Albian (although they also cautioned against too literal an interpretation of the pterosaurian fossil record because of its incompleteness). Collectively, the recent spectacular discoveries of pterosaurs from the Jehol Group have helped to generate the following palaeobiogeographical scenario: (1) an influx of older lineages (e.g. anurognathids and ctenochasmatids) from Europe to East Asia during the Barremian–Aptian (as seen in the Yixian Formation), (2) the origin of new groups such as tapejarids and advanced pteranodontoids in East Asia in the Aptian–Albian (e.g. in the Jiufotang Formation) and (3) dispersal of these new groups to South America in the Albian (e.g. Wang and Zhou 2003; Wang et al. 2005, 2007, 2012; Witton 2008). Aspects of this scenario, however, are contradicted by our current knowledge of the stratigraphical and geographical distributions of pterosaurs. In particular, anurognathids are currently known from the Middle to Late Jurassic of Asia (Barrett et al. 2008; Table 1), so their occurrence in the Early Cretaceous of Asia does not need to be explained in terms of dispersal from Europe. The phylogenetic analysis used here does not support the view that *Guidraco* and *Ludodactylus* are sister taxa: instead, the former forms a clade with *Zhenyuanopterus* and *Boreopterus* from East Asia, whereas the latter is more closely related to the anhanguerid clade that includes East Asian, European and South American taxa (Figure 2). Finally, although tapejarids might have originated in East Asia, this event would predate the Yixian Formation (see ghost range reconstructions in Figure 2), and it is possible that this clade first appeared elsewhere and only subsequently dispersed to this region. Indeed, the European tapejarid *Europejara* is late Barremian–early Aptian in age and is therefore contemporaneous with, or even predates, the earliest East Asian tapejarids (Figure 2). It could be argued that the most basal members of Tapejaridae are from East Asia and that this supports the hypothesis that this family originated in this region. However, this hypothesis cannot be tested rigorously until we obtain more tapejarid fossils from the period when this clade is likely to have originated and radiated (i.e. the early Early Cretaceous). Moreover, as with the istiodactylids (see above), we have a situation where the phylogenetic topology is consistent with the proposed dispersal event, but the stratigraphic order of taxa is not. Such incongruence suggests the occurrence of significant gaps in our current knowledge of pterosaurian distributions and argues against too literal an interpretation of the fossil record.

The Caribbean ( = ‘Hispanic’) corridor was a seaway that formed between North and South America in the Oxfordian, creating a marine connection between the western arm of Tethys and the eastern Pacific (Gasparini et al. 2004; Gasparini and Itorralde-Vinent 2006; Martill et al. 2006). Pterosaurian remains, such as *Cacibupteryx* and *Nesodactylus*, are known from the Oxfordian of Cuba, which lies in the Caribbean corridor (Gasparini et al. 2004; Gasparini and Itorralde-Vinent 2006). Martill et al. (2006) reported indeterminate ctenochasmatid material from the Early Cretaceous of Chile, and suggested that these pterosaurs might have dispersed from Europe to South America via the Caribbean corridor. Thus, the apparent increase in faunal similarity between eastern Asia, Europe and South America noted by Wang and Zhou (2003), Wang et al. (2005, 2007), Martill et al. (2006) and Witton (2008) has been linked to palaeogeographical events that occurred in the Late Jurassic and Early Cretaceous. However, there is a potential inconsistency here because some workers (e.g. Wang et al. 2005, 2007) have regarded seaways as possible geographical barriers that prevented the dispersal of pterosaurs until they were temporarily breached, whereas others (e.g. Martill et al. 2006) have viewed seaways as possible dispersal corridors. For example, the ctenochasmatids are interpreted as dispersing along the Caribbean corridor by Martill et al. (2006), whereas Wang et al. (2005, 2007) have proposed that this clade dispersed as a result of the disappearance of such ‘barriers’. One way to maintain at least partial support for both of these ideas is to postulate that particular groups of pterosaurs were affected by seaways in different ways because of their particular ecological requirements or flight abilities: to our knowledge, however, no one has proposed a detailed set of arguments to explain why rhamphorhynchids or ctenochasmatids could disperse along seaways, whereas anurognathids were purportedly prevented from crossing them.

The Nyctosauridae is a small clade of pterosaurs that were apparently endemic to North America initially during the Coniacian and Santonian, and also appear in the Maastrichtian of South America. This distribution pattern for nyctosaurids might reflect no more than the patchiness of the fossil record, but it is interesting to note that their apparent dispersal from North America to South America during the Maastrichtian is consistent with the faunal exchange hypothesis proposed by Bonaparte and Kielan-Jaworowska (1987), Lucas and Hunt (1989), Gayet et al. (1992) and Sullivan and Lucas (2000), based on the formation of a land bridge between these continents.

Buffetaut et al. (1997) noted that pterosaurs, principally azhdarchids, were still geographically widespread even during the latest Maastrichtian, and commented that this is anomalous for a group that was supposedly in decline and on the point of final extinction (see also Company et al. 1999). There is considerable evidence that larger geographical range is related to a decrease in extinction risk (Jablonski and Raup 1995; Purvis et al. 2000; Kiessling and Baron-Szabo 2004; Jablonski 2008; Purvis 2008), and it is therefore interesting to note that the last of the pterosaurs were widespread in the Maastrichtian. However, large body size is associated with increased vulnerability to extinction, especially mass extinction events (Archibald 1996; Fara 2000). Given that the last pterosaurs were mainly large animals, with wing spans typically around 4–5 m (possibly reaching up to 10 m or more), they may have been exposed to extinction risks that could not be compensated for by their wide geographical ranges and ability to move away from areas undergoing severe environmental degradation.

## 4. Analyses of pterosaurian biogeography

### 4.1 Materials and methods

#### 4.1.1 Data-set

The cladistic biogeographical analyses described below require information on pterosaurian phylogeny and the geographical and stratigraphical ranges of taxa. The reference phylogeny used in this analysis is that of Andres et al. (2014) which includes 108 terminal pterosaurian taxa ranging from the Late Triassic to the Late Cretaceous in age (Figures 1 and 2). This phylogeny is fully resolved apart from a single trichotomy linking the three terminals comprising Nyctosauridae. Cladistic biogeographical methods, such as Treefitter (see below), cannot deal with polytomies, so this trichotomy must be removed prior to analysis. All three of the nyctosaurid taxa occur in the Late Cretaceous, two in North America and one in South America (Figure 2). As this trichotomy involves just two geographical areas, we can simply resolve it into a set of bifurcating relationships without significant alteration of the biogeographical ‘signal’ in the data (i.e. all possible resolutions support a close relationship between North and South America in the Late Cretaceous). Here, therefore, we have arbitrarily resolved this trichotomy as [*Muzquizopteryx* *coahuilensis* (*Nyctosaurus* *gracilis*, *Nyctosaurus* *lamegoi*)] in the three versions of the data-set (i.e. ‘all taxa’, ‘Cretaceous taxa’ and ‘Late Cretaceous taxa’ – see below for discussion of data-set partitioning).

The geographical and stratigraphical ranges of pterosaurian taxa were obtained from Barrett et al. (2008) with some modifications based on *The Paleobiology Database* and *Fossilworks*. We have assigned each pterosaur to one or more of five areas: EA, East Asia (e.g. China and Mongolia); CA, Central Asia (e.g. Kazakhstan); EU, Europe; NA, North America; SA, South America (note that the Late Triassic *Eudimorphodon* *cromptonellus* from Greenland is here assigned to ‘Europe’ because these two areas were in close contact at this time; see Brusatte et al. 2013, Figure 7). These areas could be divided more finely: for example, we could assign South American taxa to Patagonia, Brazil and Chile. However, the current pterosaurian data-set is relatively small compared with those for other groups, such as dinosaurs, and further subdivision of areas would decrease the ability of cladistic biogeographical analyses to recover a distribution pattern common to several clades. As with the time-slicing of data (see below), the selection of areas used in a biogeographical analysis is based on the judgement of the investigators: too few areas or too many areas can render the results both biogeographically meaningless and statistically non-significant. We would need a somewhat larger data-set before further subdivision of areas could be attempted.

The rhamphorhynchids *Nesodactylus* and *Cacibupteryx* from the Oxfordian Jagua Formation of Cuba (Gasparini et al. 2004) are potentially problematic because Cuba lies in the Caribbean corridor between North and South America. Thus, these pterosaurian taxa could potentially be assigned to area NA, SA or NA+SA. Gasparini and Itorralde-Vinent (2006, p. 354) suggest that an emergent ridge stretched from Florida to the Yucatan during the Oxfordian and probably represents the source for the terrestrial fauna present in the Jagua Formation. This means that the Jagua Formation deposits were laid down on the continental margin of Laurasia and probably received North American terrestrial taxa. Therefore, in our analyses, we have provisionally assigned *Nesodactylus* and *Cacibupteryx* to area NA.

Certain terminal taxa have been pruned from some or all time-sliced data-sets because their geographical area occurs only once in that data-set. For example, *Arambourgiania* *philadelphiae* occurs in the Late Cretaceous of Jordan. The latter lies on the Arabian plate that, in the Late Cretaceous, was still connected to the rest of the African landmass (e.g. Smith et al. 1994; Scotese 2004). Although Africa has produced some fragmentary pterosaurian remains from other time periods (see Table 1), *Arambourgiania* is the only ‘African’ pterosaur in the phylogeny. Cladistic biogeographical analyses cannot produce meaningful reconstructions of the relationships of areas that occur only once in a data-set. This is because such analyses typically work by determining the level of congruence between area relationships in two or more clades (Nelson and Platnick 1981): such congruence cannot be assessed when an area occurs only once. Therefore, *Arambourgiania* has been pruned from all data-sets. Other areas, such as Central Asia (CA), occur several times in the ‘all taxa’ data-set, but occur only once in some of the time-sliced data-sets (e.g. ‘Late Cretaceous’). When time-slicing produces such ‘singleton’ areas, the relevant taxa have been deleted.

The pterosaurian biogeographical data-set has been analysed as a whole (‘all taxa’ data-set) and in a variety of different time slices (e.g. ‘Late Jurassic’ and ‘Early Cretaceous’; see Tables 2 and 3). This time-slicing approach is based on the principle that biogeographical patterns change through time in a network-like (reticulate) rather than hierarchical way (Grande 1985; Lieberman 2000; Hunn and Upchurch 2001; Upchurch and Hunn 2002; Upchurch et al. 2002; Halas et al. 2005). This means that a single biogeographical data-set might contain two or more temporally distinct, but incongruent, distribution patterns that obscure each other. Time-slicing is therefore an exploratory technique designed to search data-sets at various temporal scales to elucidate how many separate patterns exist and how these are distributed.

**Table 2:** Summary of results of Treefitter analyses based on SC and MC costs.

Time slice	No. of taxa	No. of areas	SCs	No. of SC optimal trees	SC *p*-value	MC cost	No. of MC optimal trees	MC *p*-value
Total data	106	5	52	2	0^*^	− 18	8	1.0
Late Triassic–Late Jurassic	42	4	17	1	0.0012^*^	− 7	4	1.0
Middle and Late Jurassic	19	4	10	2	0.00056, 0.00057^*^	− 6	4	0.98
Late Jurassic	20	3	3	2	0.24	− 3	3	1.0
Late Jurassic–Early Cretaceous	76	4	32	5	0^*^	− 12	2	1.0
Early Cretaceous	50	3	18	2	0.0022, 0.0029^*^	− 7	1	1.0
Cretaceous	58	4	26	3	0–0.0002^*^	− 11	1	1.0

^*^Statistically significant *p*-values.

**Table 3:** Biogeographical event frequencies.

Time slice	Vicariance	Sympatry	Extinction	Dispersal
Total data	7–10	72–75 (gtr *p* = 0)	6–12	20–23 (ltr *p* < 0.029 in one of the four area cladograms)
Late Triassic–Late Jurassic	2	31 (gtr *p* < 0.0026)	1	8
Middle and Late Jurassic	4–5	18 (gtr *p* < 0.026–0.028)	2–4	3–4
Late Jurassic	2	13	1	1
Late Jurassic–Early Cretaceous	2–8 (ltr *p* < 0.0078)	53–55 (gtr *p* = 0)	0–14 (ltr *p* < 0.012)	9–16 (ltr *p* < 0.048)
Early Cretaceous	1–2 (ltr *p* < 0.036)	37 (gtr *p* < 0.0018–0.00019)	0–2 (ltr *p* < 0.031)	8–9
Cretaceous	4–6	38–41 (gtr *p* = 0–0.0002)	4–14	6–11 (ltr *p* < 0.03 in one of the three area cladograms)

Notes: All calculations were carried out using SCs and 10,000 pterm randomisations. Only statistically significant *p*-values are listed. *p*-Values marked with ‘gtr’ indicate an event type that occurs more often than expected from random data, and those marked with ‘ltr’ indicate event types that occur less often than expected from random data.

Turner (2004) proposed a refinement to the application of time-slicing in cladistic biogeographical analyses. He noted that time-sliced cladograms may include divergence events that actually occurred prior to the time slice in question. For example, the cladogram (W (X (Y, Z))) includes three nodes. Suppose taxa W, Y and Z occur in time slice t_2_, but taxon X occurs in the earlier time slice t_1_. Time-slicing this cladogram so that it contains only taxa from t_2_ gives (W (Y, Z)), but the node representing the most recent common ancestor of W and (Y, Z) must also lie in time slice t_1_ because of the age of X. Under such circumstances, Turner's logic argues that W should be pruned from the t_2_ data-set so that the biogeographical analysis only considers divergence events that occurred in t_2_. Upchurch et al. (2002) and Turner (2004) applied Component version 2.0 (Page 1993) and Treemap (Page 1995) in order to search for biogeographical signals in their time-sliced data-sets. These methods require a single cladogram topology. This requirement means that the only way to remove divergence events that lie outside of the time slice under investigation is to prune terminal taxa from the cladogram. Fortunately, the analytical method applied here (i.e. Treefitter, see below) can search for biogeographical signals simultaneously across two or more tree topologies, which means that at least some of the phylogenetic events that lie outside of a time slice can be removed without loss of terminal taxa. For example, suppose we have a clade represented by seven taxa A–G, with the relationships ((A, (B, C)), (D, (E, (F, G)))), with D occurring in time slice t_1_ and ABCEFG occurring in t_2_. The t_2_ time-sliced cladogram contains the relationships ((A (B, C)), (E, (F, G))). The t_1_ age of taxon D means that the node representing the most recent common ancestor of (A (B, C)) and (E, (F, G)) is dated at t_1_ and should be removed from the t_2_ time slice. This can be done in Treefitter without any further deletions of terminal taxa simply by treating (A (B, C)) and (E, (F, G)) as two separate clades (rather than two sister clades in a single cladogram) in the data-set. This protocol of terminal taxon pruning and clade separation has been applied here in order to derive the time-sliced data-sets for pterosaurs.

We have not analysed the ‘Late Triassic’, ‘Early Jurassic’ or ‘Late Triassic+Early Jurassic’ time-sliced data because these contain pterosaurs from just one area (i.e. all but one of the taxa come from Europe and the singleton is from Greenland, which is treated here as part of Europe; see above). Similarly, we have not analysed the ‘Late Cretaceous’ data-set because this has multiple representations of only two areas (North America and Europe), whereas all other areas (Central Asia, East Asia and South America) occur only once each. Application of a cladistic biogeographical analysis to a data-set containing taxa from just one or two areas is not meaningful: a minimum of three areas is required in a manner analogous to the way phylogenetic analysis is only meaningful when applied to three or more taxa. Here, therefore, information on the Late Triassic, Early Jurassic and Late Cretaceous biogeographical histories of pterosaurs is derived from the analyses of the ‘all taxa’, ‘Late Triassic–Late Jurassic’ and ‘Cretaceous’ data-sets (see Tables 2 and 3).

The formatted Treefitter data files are presented in the online electronic supplement.

#### 4.1.2 Analytical protocol

Treefitter 1.2b (Apple Macintosh version) is a computerised package for biogeographical analysis (Ronquist 1998; Sanmartin and Ronquist 2004). This is an ‘event-based’ method in which each of the four following types of biogeographical events is assigned a cost: vicariance, dispersal, sympatric speciation and extinction. Given a taxon phylogeny and information on the geographical ranges of the terminal taxa, Treefitter calculates the optimal biogeographical reconstruction(s) (i.e. the biogeographical history with the minimum cost). Event costs are set by the investigator. In this study, we have employed two cost regimes. The first cost regime, known as ‘Standard costs’ (SCs), sets vicariance and sympatry at 0, extinction at 1 and dispersal at 2. The second cost regime, known as ‘Maximum Codivergence’ (MC), sets vicariance at − 1, and extinction, sympatry and dispersal at 0 (see Sanmartin and Ronquist 2004 for further discussion of Treefitter cost regimes). The MC regime mimics analyses produced by Component and TreeMap (Page 1993, 1995), which have been employed previously to search for, and test the statistical strength of, vicariance patterns in dinosaurs (Upchurch et al. 2002) and Cretaceous crocodiles (Turner 2004).

The costs assigned to each biogeographical event might seem both arbitrary and unfair in the sense that they apparently favour the discovery of vicariance patterns (see Posadas et al. 2006 for a critical discussion of ‘event costs’). However, this problem is overcome by the use of randomisation tests, which determine whether the number of each event type is greater or less than expected by chance. This approach is analogous to that used in many phylogenetic methods. A cladistic analysis will produce one or more ‘most parsimonious trees’ even if the input data are random. Before we accept a given topology as a genuine reflection of phylogenetic relationships, it is essential that we evaluate what kinds of results would be produced by random data-sets of the same dimensions and demonstrate that our real data possess a significantly greater amount of hierarchical structure (signal) than would be expected by chance. In phylogenetics, this is achieved by applying randomisation tests such as a permutation-tail probability (PTP) test (Alroy 1994; Swofford 2002). The same logic applies in the case of biogeographical analysis. Some form of optimal biogeographical reconstruction will be produced even when random data are fed to Treefitter: therefore, we can only accept that the results are meaningful biogeographical signals if it can be shown that they cannot be easily explained by chance. This means that the precise cost regime we employ is less of a concern because if we make it easier to find vicariance events in our analyses of the real data, we will also make it easier for vicariance events to occur in the randomised data. Put another way, data randomisation enables us to test the null biogeographical hypothesis, where the latter states that the spatial distributions of terminal taxa are effectively random with respect to phylogenetic relationships.

In Treefitter, the taxon cladogram topology can be randomly permuted thousands of times (‘ptree’ permutation), or the tree topology can be left unaltered and the positions of the terminal taxa can be permuted (‘pterm’ permutation), or both topology and terminal positions can be permuted. Here, we carry out both ptree and pterm permutations using 10,000 replicates each time. If the reconstruction cost for our original unpermuted data-set is less than the costs of 95% of the random data-sets, then this is accepted as a statistically significant result (i.e. *p* < 0.05). We have also used Treefitter to estimate the frequencies of the four types of biogeographical event in each of the optimal reconstructions. These frequencies can then be compared with those generated from random data-sets in order to determine whether, for example, dispersal has occurred more or less frequently than would be expected by chance. In this way, we investigate whether the spatial distributions of pterosaurs have been shaped by particular biogeographical processes.

### 4.2 Analyses and results

#### 4.2.1 Area cladograms

The total data-set and the six time slices were analysed using SC and MC cost regimes, and the resulting area cladograms were tested using 10,000 randomised replicates. As can be seen in Table 2, all of the SC analyses (except for the ‘Late Jurassic’ data-set) yielded significant results, whereas all of the MC analyses produced non-significant results.

#### 4.2.2 Event frequencies

The total data-set and each of the six time slices were analysed using SC and 10,000 pterm randomisations in order to determine the frequencies of biogeographical events for each reconstruction (Table 3). In general, the most frequent event is sympatry, followed by intermediate or low levels of dispersal, regional extinction and vicariance. All analyses (except for the Late Jurassic time slice) produced statistically significant support for elevated levels of sympatry. Most of these analyses produce no support for elevated or reduced levels of vicariance, regional extinction or dispersal: however, significantly low levels of these processes do occur in the Late Jurassic–Early Cretaceous time slice, and there are also lower than expected levels of vicariance and regional extinction in the Early Cretaceous, and dispersal in the total data-set and Cretaceous time slices.

## 5 Discussion

### 5.1 Interpretation of results

The statistically significant SC results for the ‘all taxa’ and most time slices (Table 2) are interesting because they suggest that there is some non-random signal in the pterosaurian data. However, this signal pertains to elevated levels of sympatry (see below) rather than area relationships formed in response to palaeogeographical events (i.e. vicariance produced by continental fragmentation or coherent geodispersal events prompted by removal of geographical barriers). Indeed, when statistically significant levels of vicariance, regional extinction and/or dispersal are observed, these pertain to lower than expected event frequencies (Table 3). Moreover, none of the MC analyses produced any statistically significant results, indicating a complete lack of support for the occurrence of vicariance events. Thus, with the exception of sympatry discussed below, we do not have grounds for rejecting the null biogeographical hypothesis. This means that most of the biogeographical scenarios for pterosaurs outlined earlier (e.g. origin of anurognathids and pterodactyloids in Central and/or East Asia as a result of Middle and Late Jurassic isolation, dispersal of rhamphorhynchids and ctenochasmatids from Europe to South America via the Caribbean corridor, and the origin of clades such as tapejarids in East Asia during the Early Cretaceous) must be regarded as speculative. Such speculations represent valuable contributions to our understanding of pterosaur biogeography insofar as they provide explanatory hypotheses that can be tested by the type of analyses applied here and also by future discoveries of new material. Nevertheless, as long as the null hypothesis remains unrejected, it must be accepted that such explanatory hypotheses might be no more than narratives created by weaving together random data points into appealing scenarios.

It is clear that pterosaurs do not display the strongly statistically significant area relationships found among dinosaurs (Upchurch et al. 2002; Upchurch 2006, 2008), Gondwanan crocodyliforms (Turner 2004) and Cretaceous terrestrial vertebrates generally (Ezcurra and Agnolin 2012). The many reasons why pterosaurs might not display statistically significant area relationships and conform to our knowledge of Mesozoic palaeogeography fall into two broad categories. First, it is possible that pterosaurs actually displayed strong biogeographical patterns such as vicariance, but these signals cannot be retrieved at present because of problems with the available data. Such problems could include taxonomic and phylogenetic errors, incorrect selection of area units, missing data or even biased sampling of the fossil record (see below). The second possibility is that our results represent a genuine reflection of pterosaur biogeographical history: that is, there is no area relationships signal in the data because there was never one to detect in the first place. The most obvious potential cause of ‘no vicariance signal’ is that the flight abilities of pterosaurs meant that they could disperse across the geographical barriers that controlled the distributions of terrestrial organisms during the Mesozoic. As Unwin (1996, p. 300) stated: ‘It is doubtful whether pterosaurs were hindered by most natural obstacles, such as mountains or seas.’ Given the information and results to hand, both of these types of explanation are equally valid, although we note that none of the event frequency analyses produced any statistical support for more dispersal than would be expected from random data, and in fact some analyses indicate lower than expected levels of dispersal.

What then is the meaning of the higher than expected levels of sympatry throughout much of the pterosaur data-set? First, it should be noted that ‘sympatry’ in a Treefitter analysis simply refers to a duplication event (i.e. a phylogenetic lineage living in area X diverges into two daughter lineages that also occur in area X). Duplication events might represent true sympatry (i.e. speciation caused by populations specialising for life in different habitats within the same geographical region) or ‘within-area allopatry’ (WAA) (i.e. speciation caused by the formation of a barrier to dispersal within the designated geographical region) (see Xu et al. 2013). Although it is extremely difficult to tease apart genuine sympatry from allopatry in the fossil record, there is some circumstantial evidence that pterosaur duplication events often relate to the former process. Pterosaur Lagerstätten in the Early Cretaceous of China and Brazil demonstrate that many coeval species apparently inhabited the same environments and had overlapping geographical ranges. For example, the Romualdo Formation (early Albian) of the Santana Group, Ceará, Brazil, has produced four species of *Anhanguera* and a further five pterosaur genera such as *Tropeognathus* and *Cearadactylus* (based on data from *The Paleobiology Database*). This provides *prima face* evidence that several of the duplicated pterosaur lineages were not partitioned geographically as predicted by the WAA explanation (see comments on chronospecies and time-averaging below).

Before attempting an evolutionary explanation of the elevated levels of sympatry, it is important to consider the extent to which these results might be artefactual. One possibility is that the high levels of sympatry have been produced by uneven sampling of the fossil record. Suppose, for example, that clade A has members that mainly inhabit inland freshwater environments, whereas members of clade B occur largely in coastal habitats. Suppose also that, during a given time interval, both clades are distributed across the same set of areas. If geological or anthropogenic factors mean that we mainly sample coastal sediments from continent X and inland ones from continent Y, we will find that clade A has many closely related species ‘endemic’ to Y and clade B has many closely related species ‘endemic’ to X. Such a pattern would mimic the effects of sympatry or WAA and might be sufficient to produce statistically significant levels of support in a Treefitter analysis. This phenomenon might be a significant issue for the results based on data from the Early Cretaceous. The pterosaurs from the Barremian of Europe and Aptian of China are known largely from continental deposits, whereas those from the Albian Santana Group of Brazil are generally large-bodied forms found in lagoonal sediments. Quantitative analyses of the type applied to the distributions of non-avian dinosaurs (Butler and Barrett 2008; Mannion and Upchurch 2010a) and Mesozoic birds (Brocklehurst et al. 2012) could be used to assess the extent to which pterosaur distributions have been distorted by differential sampling of different types of environment, but lie outside the scope of the current study. One counter-argument to the sampling-bias scenario outlined above is that many deposits (especially Lagerstätten) have yielded species from several different portions of the pterosaur evolutionary tree rather than unique endemic clades. For example, the Aptian of China and the Albian of Brazil both include anhanguerids, ornithocheirids and tapejarids, despite their apparently dissimilar depositional settings (Table 1, Figures 1 and 2). Moreover, some of the small clades of pterosaurs have representatives in two or more geographical regions (e.g. istiodactylids in Europe and East Asia, and tapejarids in these two areas and South America and Africa). These distributions are inconsistent with the artefactual generation of endemic clades as a result of the uneven sampling of habitats with respect to geographical region. Nevertheless, it would be premature to argue that uneven sampling of the fossil record has played no role in artefactually boosting the apparent biogeographical signal.

A second possible cause of an artefactual biogeographical signal supporting sympatry or WAA concerns problems with alpha-level taxonomy. In particular, it is conceivable that taxonomic over-splitting could create clusters of apparently closely related species that occur in the same restricted geographical areas. Such clusters might be identified in cladograms as sets of species that form poorly resolved clades (since there would often be no hierarchical character data available to separate them into fully resolved clades). However, identification of different ontogenetic stages of a single species as multiple species, or the occurrence of time averaging within deposits so that separate chronospecies appear to be contemporaneous, could potentially generate hierarchically distributed character data that would result in fully resolved species clusters in cladograms. For example, the tapejarids *Nemicolopterus*, and *Sinopterus* *gui* are based on juvenile specimens (see Andres and Myers 2013), are sister taxa in the reference phylogeny (Figure 2) and have identical geographical and stratigraphical ranges: it is therefore conceivable that these two genera and perhaps others from the Aptian of China have been diagnosed on the basis of ontogenetic variation rather than apomorphies that correctly indicate cladogenetic events. We acknowledge this issue as a potential problem for the currently available data for pterosaurs, but note that this clade is not unique in this respect. Such taxonomic problems are a perennial issue for all palaeobiological studies that depend on phylogenetic topologies for their quantitative and/or statistical rigour. Taxonomic revision of pterosaurs lies outside of the scope of the current study and we suggest that, while caution is advisable, such issues do not preclude the interpretation of our results as genuinely supporting sympatry. This is a working hypothesis that can easily be overturned by future discoveries of new taxa, revisions of pterosaur taxonomy and further analysis of phylogenetic relationships.

The high levels of sympatry and low levels of dispersal and vicariance within the pterosaur data-sets support a new hypothesis for the biogeographical history of this clade. The powered flight of pterosaurs might have enabled certain lineages to occasionally cross-geographical barriers such as wide oceans and mountain ranges. However, such events were apparently comparatively rare in pterosaur evolution (though frequent enough to overprint any vicariance signals generated by Pangaean fragmentation and fluctuations in sea level). The rarity of successful dispersal across geographical barriers might relate to ecological rather than locomotor requirements: that is, pterosaurs could have found it relatively easy to fly over a barrier, but might have had difficulties in founding viable populations once they reached anew area because of differences in food sources or other ecological parameters. On those rare occasions when pterosaur lineages successfully dispersed into new regions, they apparently tended to diversify within those areas, perhaps specialising to a variety of different niches defined by body size, feeding preferences/strategies and perhaps habitat types. Such clusters of sympatric pterosaur taxa can be detected in the data-set because dispersal across barriers was apparently not frequent enough to overprint these patterns. This view is supported by the observation that, despite their volant abilities, very few pterosaur sister taxa or species have widespread geographical distributions (see Barrett et al. 2008; *The Paleobiology Database*; *Fossilworks*). Thus, pterosaur biogeographical history may be characterised as a series of occasionally successful ‘sweepstakes’ dispersal events, several of which led to regionally restricted sympatric radiations. If correct (and putting aside sampling biases and taxonomic over-splitting for the present), apparently endemic pterosaur radiations (such as the Lonchodectidae in Europe and Pteranodontidae in North America) were geographically restricted because of their specialised ecological requirements rather than an inability to cross-geographical barriers.

### 5.2 Fossil record quality and biogeography

The description of pterosaurian biogeographical history outlined above illustrates some common problems in palaeobiogeography. For example, palaeobiologists frequently assume that the area that has produced the earliest member of a given clade represents the ‘centre of origin’ of that clade (e.g. the previously proposed origin of tapejarids in the Aptian of East Asia). Furthermore, when that clade is found in other areas later in the stratigraphical record, this is interpreted as evidence for dispersal from the centre of origin. Such scenarios are legitimate explanations of the data, but they are not the only viable ones. The same observed distributions could also be created by a combination of vicariance and missing data. For example, consider Wang and Zhou's (2006) suggestion that tapejarid pterosaurs originated in the Barremian–Aptian of China, based on the observation that the earliest members of this clade were known at that time from the Yixian Formation. The subsequent appearance of tapejarids in the Albian Santana Group in Brazil is therefore interpreted as evidence for dispersal from China to South America. However, it is also conceivable that tapejarids originated long before the Barremian and achieved a widespread or even global distribution. Under this second hypothesis, the presence of tapejarids in China and South America would be the product of imposing a Lagerstätten effect on a global distribution. Such a scenario implies a somewhat earlier origin for tapejarids, potentially as early as the Middle Jurassic separation of Laurasia from Gondwana. Palaeobiologists frequently reject such ideas because they imply an unacceptably large amount of missing fossil record. Thus, competing interpretations of pterosaur biogeography are bound up with workers' implicit beliefs about the quality of the group's fossil record. Any tendency to minimise the assumed amount of missing data will increase the probability of devising a dispersal-based explanation for the observed geographical distributions in the fossil record. Clearly, quantifications of missing data and sampling biases have a key role to play in analytical biogeographical analyses as they do in diversity reconstruction.

The issue of the quality of the pterosaur fossil record has received some attention recently, especially with regard to diversity. Dyke et al. (2009) carried out a number of analyses, including evaluation of the congruence between phylogeny and stratigraphy, in order to examine whether the pterosaur fossil record is adequate for macroevolutionary studies. These authors concluded that the pterosaur fossil record is indeed adequate for such studies and that there is no ‘Lagerstätten effect’ (i.e. distortions created by rare examples of exceptionally rich fossil deposits, such as the Jehol Group biotas). In contrast, Butler et al. (2009, 2013) examined the extent to which pterosaur diversity correlates with a proxy for sampling intensity (the number of pterosaur-bearing formations through time) and argued that many of the observed fluctuations in diversity are sampling artefacts closely linked to Lagerstätten occurrences. The results presented here cannot decisively settle this issue because statistical failures can be explained in terms of errors, missing data, sampling biases and so on, or as real reflections of a biogeographical history dominated by one-off dispersal events. Moreover, we suggest that it is often meaningless to categorise the fossil record of a given group as either entirely ‘adequate or ‘inadequate’ – in many cases a group's record is good enough for some types of macroevolutionary study and too incomplete or unevenly sampled for others. The question palaeobiologists need to address, therefore, is: ‘Is the fossil record of this group adequate for the study of a particular aspect of evolutionary history?’ Below we elaborate on this point by briefly considering some aspects of pterosaurian macroevolution in the light of their phylogenetic relationships, fit to stratigraphical order and palaeogeographical distributions.

Figures 1 and 2 support Dyke et al.’s suggestion that there is a high degree of congruence between the order of appearance of pterosaurs in the fossil record and the branching structure of their phylogenetic relationships. This indicates that the relative order of appearance of pterosaur clades probably reflects genuine evolutionary history rather than uneven sampling. Although there are uncertainties regarding the exact timing of such events, it seems reasonable to suggest that a clade of *Eudimorphodon*-like taxa radiated during the Late Triassic but became extinct at or near the Triassic–Jurassic boundary. Similarly, a disproportionate number of lineages apparently terminate at the Jurassic–Cretaceous boundary, followed by the radiation of new lineages in the early Cretaceous. Such a pattern mirrors that observed in several dinosaurian groups (Barrett et al. 2009; Mannion et al. 2011; Upchurch et al. 2011) and marine reptiles (Benson et al. 2010), and supports the hypothesis of a major extinction at the Jurassic–Cretaceous boundary (Upchurch and Mannion 2012). Finally, Figure 2 indicates a possible mid-Cretaceous faunal turnover event among pterosaurs (see also Butler et al. 2012, 2013) that potentially parallels that seen among dinosaurs, crocodiles, mammals and squamates (Benson et al. 2013; see the caveat concerning the relative scarcity of Lagerstätten in the Late Cretaceous noted earlier in Section 2.5). However, although the pterosaur fossil record is apparently good enough to enable reconstructions of the broad outlines of radiations and extinction events, this does not mean that the magnitude and direction of diversity change are reliable. As noted by Butler et al. (2009, 2013), observed pterosaurian diversity is strongly correlated with estimates of sampling, and the highest peaks in diversity coincide precisely with the occurrences of Lagerstätten.

The impact of Lagerstätten on the proposed biogeographical histories of pterosaurs can also be observed clearly in our data-sets. For example, as noted above, several authors have commented on the apparent close biotic similarity of the Aptian Jehol and Albian Santana faunas, resulting in the suggestion that the former acted as a centre of origin and that dispersal from East Asia to South America (perhaps via Europe) occurred at this time. At present, the Early Cretaceous time slice only contains pterosaurs from three areas, Europe, East Asia and South America, the two latter regions being strongly represented largely because their Lagerstätten deposits have yielded enough pterosaurs of sufficient quality for them to be incorporated into phylogenetic analyses. Yet, Table 1 indicates that pterosaurs were actually globally distributed during the Early Cretaceous, but forms from Africa, Australia and so on have not been added to phylogenies, perhaps reflecting poor preservation of the available material. We cannot produce a meaningful test of the proposed Early Cretaceous hypotheses for pterosaur biogeography until we have adequate samples from other key areas such as Africa, North America and parts of east Gondwana. We conclude, therefore, that the frequently noted similarity between the Aptian East Asian and Albian South American pterosaur faunas is likely to be an artefact created by the presence of Lagerstätten – in effect, the fossil record from other regions is too poor to provide adequate comparisons.

Finally, Table 1 provides a crude estimate of the spatiotemporal sampling of the pterosaurian fossil record. This table is divided into 26 stratigraphical stages and 9 geographical areas, giving a total of 234 cells. Of these, 59% are empty, and this rises to 66% when cells that contain only records of indeterminate pterosaur material are also considered empty. Some of the empty cells potentially reflect true absences: for example, if pterosaurs genuinely radiated in Euramerica during the Late Triassic, then absence in Gondwana and Central and East Asia from the Carnian through to one or more of the Early Jurassic stages would reflect real absence rather than poor sampling. Nevertheless, this simple measure suggests that the pterosaurian fossil record is very patchy both spatially and temporally. As well as supporting the conclusions of Butler et al. (2009, 2013) regarding pterosaur diversity, these data also argue for considerable caution when attempting to reconstruct the biogeographical history of this group.

## 6. Conclusions and future prospects

Pterosaurs have proved to be an excellent model system for studies of vertebrate biomechanics (notably powered flight), but their current potential for other types of macroevolutionary analysis is questionable. A direct reading of the pterosaur fossil record suggests that this group rapidly achieved a global distribution in the Early Jurassic, and that subsequent radiations may have been restricted to particular areas (e.g. anurognathids in Laurasia), or dispersed widely (e.g. azhdarchids). However, literal interpretations of the fossil record are dangerous because they do not take sampling biases into account, and do not attempt to reject the null biogeographical hypothesis. Our analyses suggest that there is no convincing statistical support for area relationships among pterosaurs or for the dominance of particular types of biogeographical processes such as vicariance or dispersal. There is, however, evidence for elevated levels of sympatry over much of pterosaurian evolutionary history, potentially indicating a combination of rarely successful sweepstakes dispersal events across barriers and subsequent regional radiations among the founding populations of these dispersers.

The almost complete lack of vicariance and dispersal signals in the pterosaur data is disappointing, but it should be remembered that this situation could change radically in the near future. At least four important lines of further enquiry can be identified. First, as always, new discoveries have the potential to improve the quality of sampling in our data-sets, although it should also be noted that there are many currently known pterosaur remains that could be productively integrated into phylogenetic analyses. Second, the study of palaeoecology (including analyses of associations between clade occurrences and different facies types), flight biomechanics and physiology need to be integrated to provide models of the different ecological requirements and dispersal abilities of pterosaurs. Whether or not a particular geographical feature (such as a seaway or climatic zone) represents a barrier to dispersal or a dispersal corridor might depend on which type of pterosaur is involved. There is every possibility, for example, that a wide seaway that represented a considerable barrier to small pterosaurs might be crossed easily by forms with larger wingspans. Similarly, such a seaway might have provided a convenient dispersal corridor for taxa that depended on marine organisms for their diet, but might also have severely limited the range of those pterosaurs that obtained food principally from terrestrial sources. Thus, some parts of the pterosaur data-set might contain strong support for non-random area relationships, whereas others might be indistinguishable from random. Third, the geological and anthropogenic factors that potentially control the sampling of the pterosaur fossil record need further investigation. For example, application of the completeness-metric approach proposed by Mannion and Upchurch (2010b) and Brocklehurst et al. (2012), and analyses of which pterosaur clades occur in which facies, should provide insights into the extent to which absence in the fossil record indicates genuine absence or missing data. Finally, it would be interesting to examine how pterosaur biogeography compares with any spatial patterns in the other Mesozoic vertebrate clade that possessed powered flight – birds. In the meantime, this study provides the first quantitative analysis of pterosaurian biogeography, and it is hoped that it will therefore serve as a foundation for more detailed studies in the future.
